# A minimal PBPK model to accelerate preclinical development of drugs against tuberculosis

**DOI:** 10.3389/fphar.2023.1272091

**Published:** 2024-01-04

**Authors:** Federico Reali, Anna Fochesato, Chanchala Kaddi, Roberto Visintainer, Shayne Watson, Micha Levi, Véronique Dartois, Karim Azer, Luca Marchetti

**Affiliations:** ^1^ Fondazione The Microsoft Research—University of Trento Centre for Computational and Systems Biology (COSBI), Rovereto, Italy; ^2^ Department of Mathematics, University of Trento, Povo, Italy; ^3^ Gates Medical Research Institute, Cambridge, MD, United States; ^4^ Hackensack Meridian Health, Nutley, NJ, United States; ^5^ Department of Cellular, Computational and Integrative Biology (CIBIO), University of Trento, Povo, Italy

**Keywords:** minimal PBPK model, mPBPK, antituberculosis agents, modeling, simulation, tuberculosis, model informed drug development

## Abstract

**Introduction:** Understanding drug exposure at disease target sites is pivotal to profiling new drug candidates in terms of tolerability and efficacy. Such quantification is particularly tedious for anti-tuberculosis (TB) compounds as the heterogeneous pulmonary microenvironment due to the infection may alter lung permeability and affect drug disposition. Murine models have been a longstanding support in TB research so far and are here used as human surrogates to unveil the distribution of several anti-TB compounds at the site-of-action via a novel and centralized PBPK design framework.

**Methods:** As an intermediate approach between data-driven pharmacokinetic (PK) models and whole-body physiologically based (PB) PK models, we propose a parsimonious framework for PK investigation (minimal PBPK approach) that retains key physiological processes involved in TB disease, while reducing computational costs and prior knowledge requirements. By lumping together pulmonary TB-unessential organs, our minimal PBPK model counts 9 equations compared to the 36 of published full models, accelerating the simulation more than 3-folds in Matlab 2022b.

**Results:** The model has been successfully tested and validated against 11 anti-TB compounds—rifampicin, rifapentine, pyrazinamide, ethambutol, isoniazid, moxifloxacin, delamanid, pretomanid, bedaquiline, OPC-167832, GSK2556286 - showing robust predictability power in recapitulating PK dynamics in mice. Structural inspections on the proposed design have ensured global identifiability and listed free fraction in plasma and blood-to-plasma ratio as top sensitive parameters for PK metrics. The platform-oriented implementation allows fast comparison of the compounds in terms of exposure and target attainment. Discrepancies in plasma and lung levels for the latest BPaMZ and HPMZ regimens have been analyzed in terms of their impact on preclinical experiment design and on PK/PD indices.

**Conclusion:** The framework we developed requires limited drug- and species-specific information to reconstruct accurate PK dynamics, delivering a unified viewpoint on anti-TB drug distribution at the site-of-action and a flexible fit-for-purpose tool to accelerate model-informed drug design pipelines and facilitate translation into the clinic.

## Introduction

With a claim of 1.6 million lives and 10.6 million new infected cases in 2021 (World Health Organization, 2022), tuberculosis (TB) is among the leading global-health issues of modern days. The historical standard of care treatment, as well as the latest FDA-approved regimens against resistant strains, are highly intensive, with scarce patient compliance and severe adversary effects reported (Gillespie et al., 2014; Jindani et al., 2014). Therefore, there is an urgent need for innovation to shorten anti-TB regimes, without compromising safety and efficacy criteria, to finally increase the success rate and alleviate the toll placed on individuals and the healthcare system (World Health Organization, 2022).

In this sense, although no true animal reservoirs for *Mycobacterium tuberculosis* exist (Dharmadhikari and Nardell, 2008), several preclinical organisms are found susceptible to the TB infection and thus used for investigative TB studies. To date, animal models of active pulmonary TB have remarkably enhanced our understanding of TB pathogenesis, host/pathogen interaction, and immune system contribution, as well as guided the design of antimicrobial regimens (Dharmadhikari and Nardell, 2008). Common thread of these preclinical contributions is the rich collection of measurements at both systemic and peripheral tissues that have served as soil for generating hypothesis on either drug or disease, supplementing the paucity and sparsity of intra-pulmonary clinical data. In this regard, PK samples in animal lungs combined with *in silico* predictive technologies are instrumental to unveil anti-TB drug attainment in *Mycobacterium* habitat, questioning the paradigm of considering plasma exposure as a surrogate of TB site-of-action levels.

At present, most of these computational PK suites are tailored to each compound of interest; hence they may lack a systems viewpoint across drug classes and investigators. The specificity of these model-design choices, which are grounded on the expertise of the modeler in balancing qualitative assessments and quantitative estimations, can hamper unbiased drug comparisons and reproducibility. These tools leverage pharmacokinetic (PK), population pharmacokinetic (popPK), and physiologically-based pharmacokinetic (PBPK) approaches, and have proved to be exceptional virtual labs to characterize and quantify drug absorption, distribution, metabolism, and excretion (ADME) processes involved in the intricate TB pathology (Lyons et al., 2013; El-Khateeb et al., 2021; Humphries et al., 2021). PK and popPK models are well suited for easy-to-get and easy-to-read prediction of PK parameters and for covariate identification, but their *top-down* nature lacks a biological counterpart as species- or drug-specific information is not accounted for (Jones and Rowland-Yeo, 2013). In contrast, PBPK frameworks are appreciated for capturing mechanistic and biochemically-informed PK profiles based on prior knowledge e.g., blood flow distributions or transport processes, but they come at the cost of a higher number of parameters and longer development and simulation time (Gerlowski and Jain, 1983; Läer and Khalil, 2011; Shebley et al., 2018). As an intermediate technique, simplified versions of PBPK models are increasingly gaining momentum to reduce the dimensionality of full-body multi-compartment PBPK, while retaining reliable physiological attributes (Bloomingdale et al., 2021; Yau et al., 2023). Following the law of parsimony, these models lump tissues and organs according to suitable metrics, such as dynamical similarities or steady-state partitioning, to generate a more tractable analysis and to facilitate parameter estimation. We refer to (Coxson and Bischoff, 1987; Bernareggi and Rowland, 1991; Nestorov et al., 1998; Dokoumetzidis and Aarons, 2009; Pilari and Huisinga, 2010; Lin et al., 2016; Yau et al., 2023) for overviews of the available lumping strategies.

In the present study, we propose a data-driven minimal PBPK model (mPBPK) in mice that can simultaneously support several anti-TB drugs by focusing on transversal ADME and TB-hallmark features. As only one out of four literature-available mice strains here included—BALB/c, Swiss-derived, C57BL/6, and C3HeB/FeJ - can develop human-like lesions, the model treats the pulmonary compartment as a general site-of-action for TB. The work seeks to deliver a unified viewpoint and standardized protocol to compare candidates on desirable preclinical plasma and lung PK metrics. We have benchmarked the novel design on literature-available PK datasets of eleven drugs, both marketed—isoniazid (INH), pyrazinamide (PZA), ethambutol (EMB), moxifloxacin (MOX), rifampicin (RIF), rifapentine (RPT), delamanid (DEL), pretomanid (PRE), and bedaquiline (BDQ)—and in clinical development—GSK2556286 (G286) and OPC-167832 (OPC). Through the calibrated model, we query the optimality of standard workflows for drug-sorting administration and drug target attainment at the site-of-action.

## Materials and methods

### mPBPK model design and assumptions

**FIGURE 1:**
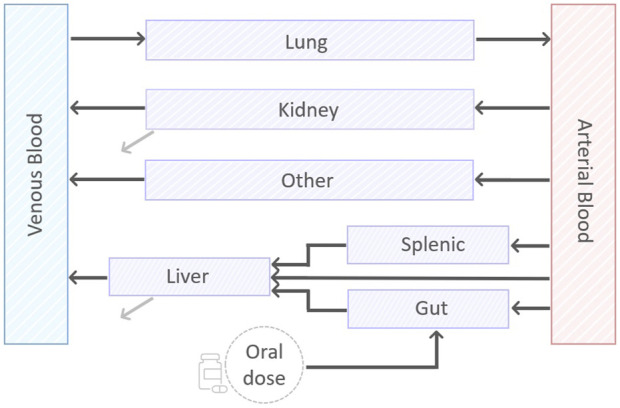
A visual representation of the minimal PBPK model. The model consists of nine compartments, eight of which describing: arterial and venous blood, gut, splenic, liver, lung, kidney, and the lumped compartment “other”. In addition, there is a compartment to account for the oral dose disposition. Black lines represent exchange between the compartments. Grey lines represent the first-order clearance.

We designed a minimal mathematical framework that depicts drug disposition across the murine body through a set of nine ordinary differential equations (ODEs). A visual representation of the minimal model can be found in Figure 1. The model results from an iterative application of the lumping strategy, as detailed in (Nestorov et al., 1998; Ryu et al., 2022), to reduce the computational complexity and increase the focus on the tissue of interest pertaining to the drug’s anticipated mechanism and sites of action. At each step, parallel connected tissues not involved in ADME processes—namely, adipose, bone, brain, heart, muscle, gonads, and skin—were incrementally lumped and the reduced model was validated via a visual predictive comparison with the original model outputs. The lumping criterium was data-driven and applied to the above TB-unessential tissues and organs regardless of their perfusion rates, body proximity, or functionality (Ryu et al., 2022). The connections involving the liver, spleen and pancreas were pooled into the splenic compartment to simplify the chain of compartments. Model simulations supported the definition of a lumped compartment, named “other”, that summarizes all eight tissues, allowing for a 77% reduction in the number of physiological variables in the ODE system (Stader et al., 2019). First and second-order kinetics for oral absorption, total clearance, compartment outflows, and possible gut-mediated reabsorption were evaluated during model development via goodness-of-fit (GOF).

In the final model (Figure 1), the dynamics of drug concentration in the arterial and venous circulatory system are described by Eqs 1 and 2.

$$\frac{dC_{ab}}{dt}=\frac{1}{V_{ab}}\left(Q_{lu}\cdot \frac{C_{lu}}{{Kp}_{lu}}\cdot BP-\;Q_{lu}\cdot C_{ab}\right)$$

Equation 1. Differential equation describing the drug concentration in the arterial blood. Since the blood is assumed to flow in and out the lungs Q_lu_ = Q_sp_ + Q_ha_ + Q_gu_ + Q_ki_ + Q_ot_.

$$\frac{dC_{vb}}{dt}=\frac{1}{V_{vb}}\left(Q_{ot}\cdot \frac{C_{ot}}{{Kp}_{ot}}\cdot BP+Q_{ki}\cdot \frac{C_{ki}}{{Kp}_{ki}}\cdot BP+Q_{li}\cdot \frac{C_{li}}{{Kp}_{li}}\cdot BP-Q_{lu}\cdot C_{vb}\right)$$

Equation 2. Differential equation describing the drug concentration in the venous blood.

where BP is the blood-to-plasma ratio, V_ab_ and V_vb_ are arterial and venous blood volumes, Q_lu_, Q_ot_, Q_ki_, Q_li_ and Kp_lu_, Kp_ot_, Kp_ki_, Kp_li_ are, respectively, the blood flow and the tissue-to-plasma partition coefficient for lung, lumped compartment, kidneys, and liver. Since all blood was assumed to flow in and out the lungs, the relationship Q_lu_ = Q_sp_ + Q_ha_ + Q_gu_ + Q_ki_ + Q_ot_ holds to fulfill mass-conservation law, with Q_sp_, Q_ha_, and Q_gu_ blood flows for splenic, hepatic artery, and gut compartments (see Figure 1).

Drug dynamics within non-eliminating compartments - lung, lumped, spleen, and gut -, is governed by the difference in drug uptake and output terms, which are driven by blood flows and partition coefficients, respectively. The corresponding equations are therefore Eqs 3–6.

$$\frac{dC_{lu}}{dt}=\frac{1}{V_{lu}}\left(Q_{lu}\cdot C_{vb}-Q_{lu}\cdot \frac{C_{lu}}{{Kp}_{lu}}\cdot BP\right)$$

Equation 3. Differential equation describing the drug concentration in the lung compartment.

$$\frac{dC_{ot}}{dt}=\frac{1}{V_{ot}}\left(Q_{ot}\cdot \left(C_{ab}-\frac{C_{ot}}{{Kp}_{ot}}\cdot BP\right)\right)$$

Equation 4. Differential equation describing the drug concentration in the “other” compartment.

$$\frac{dC_{sp}}{dt}=\frac{1}{V_{sp}}\left(Q_{sp}\cdot \left(C_{ab}-\frac{C_{sp}}{{Kp}_{sp}}\cdot BP\right)\right)$$

Equation 5. Differential equation describing the concentration in the spleen compartment.

$$\frac{dC_{gu}}{dt}=\frac{1}{V_{gu}}\left(Ka\cdot \;C_{or}\cdot F+Q_{gu}\cdot \left(C_{ab}-\frac{C_{gu}}{{Kp}_{gu}}\cdot BP\right)\right)$$

Equation 6. Differential equation describing the concentration in the gut compartment.

where V_sp_, V_gu_ are the volumes and Kp_sp_, Kp_gu_ are the partition coefficients for splenic and gut, respectively, while Ka and F are the rate of absorption and the drug bioavailability.

Observe that Equation 3, the first term of Equation 1, and the last term of Equation 2 model the pulmonary loop with the arterial and venous bloodstream, while Equation 6 reflects the oral route of administration. Indeed, an oral dose compartment with exponential decay and with full release into the gut compartment was included to mimic the oral ingestion and described in Equation 7.

$$\frac{dC_{or}}{dt}=-\left(Ka\cdot C_{or}\cdot F\right)$$

Equation 7. Differential equation describing the concentration in the oral dose compartment.

Tissues involved in drug elimination processes, liver and kidneys, present a negative term in the differential equation that specifies hepatic or renal clearance as in the following Eq. 8 and Eq. 9.

$$\frac{dC_{li}}{dt}=\frac{1}{V_{li}}\left(Q_{ha}\cdot C_{ab}+Q_{gu}\cdot \frac{C_{gu}}{{Kp}_{gu}}\cdot BP+Q_{sp}\cdot \frac{C_{sp}}{{Kp}_{sp}}\cdot BP-\;Q_{li}\cdot \frac{C_{li}}{{Kp}_{li}}\cdot BP-CL\cdot \left(1-{CL}_{R}\right)\cdot C_{li}\cdot {fu}_{p}\right)$$

Equation 8. Differential equation describing the concentration in the liver compartment.

$$\frac{dC_{ki}}{dt}=\frac{1}{V_{ki}}\left(Q_{ki}\cdot \left(C_{ab}-\frac{C_{ki}}{{Kp}_{ki}}\cdot BP\right)-CL\cdot {CL}_{R}\cdot C_{ki}\cdot {fu}_{p}\right)$$

Equation 9. Differential equation describing the concentration in the kidney compartment.

Here, CL is the total body clearance, CL_R_ is the renal clearance fraction, and fu_p_ is the fraction unbound in plasma. Note that, as plasma contributes most to the blood protein component, fu_p_ was assumed to be a good approximator of blood free-fraction and used in Eq. 8 and Eq. 9 to scale clearance outflows, capturing the part of the drug that can be eliminated.

The model was implemented in Matlab R2022b (The MathWorks Inc., 2022) and simulated via odes15 function to generate all the simulations contained in the manuscript. The included plots have been produced in Matlab and R 4.2.0.

### Model physiological and physicochemical parameters

The mPBPK model includes 25 parameters, divided into physiological information and drug physicochemical properties (see Supplementary Table S1 and Lee et al., 2020). The distribution volumes and specific blood flow rates refer to 0.025 kg representative mice and were taken from the literature (Brown et al., 1997; Ruark et al., 2014; Lee et al., 2020). Partition coefficients were calculated via the *in silico* Rodgers and Rowland (RR) equations (Rodgers, Leahy, and Rowland, 2005; Rodgers and Rowland, 2006), according to the chemical type of the drug under investigation (neutral, monoprotic acid or base, diprotic acid or base, zwitterions). For the lumped compartment, which has no physiological counterpart, we set the volume and the blood flow as the sum of those lumped in generating it (Nestorov et al., 1998), and the partition coefficient as their median. Drug-specific chemical properties required for the RR method—the octanol:water partition coefficient (clogP), the vegetable oil:water partition coefficient (logD), the negative log10 of disassociation constants (pKa1, pKa2), affinity constant, the intracellular pH of red blood cells, the blood-to-plasma ratio (BP), and the free fraction in plasma (fu_p_)—were extracted from the literature, internal sources, and DrugBank. Supplementary Table S1 contains the full set of drug-related parameters, PK information, *in vitro* pharmacodynamic (PD) metrics, and references to the sources. Complementary physiological information on the fractional tissue volumes of extra-, intra-cellular water, neutral lipids, and neutral phospholipids and the tissue concentration of acidic phospholipids, extracellular albumin, or lipoprotein came from (Ruark et al., 2014). When preclinical data regarding tissue penetration, particularly in infected tissues, were available, the corresponding RR-predicted partition coefficients were adjusted to better match *in vivo* tissue-to-plasma concentration ratio in accordance with the literature protocol (Bruzzese et al., 2000; Lyons et al., 2013; Ezuruike et al., 2022; Ryu et al., 2022). For the drugs for which no mice time series or aggregated lung data is available, we adjusted the plasma-to-lung RR-predicted partition coefficients exploiting literature interspecies ratios (Ezuruike et al., 2022). GSK2556286 is the only compound for which lung data is completely not available due to its early developmental stage, thus no refinement to the RR predictions was applied.

### Data

The model was trained and validated on literature data retrieved from published articles and datasets for 11 anti-TB compounds, - rifampicin (RIF, R), rifapentine (RPT, P), pyrazinamide (PZA, Z), ethambutol (EMB, E), isoniazid (INH, H), moxifloxacin (MOX, M), delamanid (DEL), pretomanid (PRE, Pa), bedaquiline (BDQ, B), OPC-167832 (OPC), GSK2556286 (G286). Literature PK experiments were conducted on TB infected murine models for BDQ, PZA, RIF, INH, EMB, and G286. The remaining compounds (RPT, MOX, DEL, PRE, and OPC) are based on the literature on uninfected mice PK assumed to show limited differences in terms of exposure with infected mice.

Data types spanned from aggregated PK information—area-under-the-curve (AUC), concentration peak (Cmax), time of peak (Tmax)—to sparse or dense time series. In this latter case, if PK time series were not tabulated within the publications, they were digitalized using WebPlotDigitizer (Rohatgi, 2022). Supplementary Table S2 summarizes the data sources used to develop and test the model. Plasma or serum PK measurements were multiplied by BP to obtain observations in venous blood and suit model structure for the estimation step. The tissue density to convert drug tissue concentration from µg/g to µg/mL as for plasma was set to 1 g/cm^3^.

### Parameters identification, calibration, and uncertainty quantification

Out of the 25 model parameters, the rate of absorption Ka and the total clearance CL only were calibrated from mouse PK data. A structural identifiability investigation was run on these two parameters with the Matlab toolbox GenSSI (Chiş et al., 2011) to ensure that the minimality of the framework had mitigated the general PBPK model identifiability issues (Läer and Khalil, 2011; Shebley et al., 2018; Shebley et al., 2018; Peters and Dolgos, 2019; Peters and Dolgos, 2019).

A Matlab implementation of the Covariance Matrix Adaptation—Evolution Strategy (CMA-ES) method, a state-of-the-art evolutionary algorithm (Hansen N, 2006; Auger and Hansen, 2012; Hansen, 2016), was used for calibrating the model. The optimization algorithm was initialized considering a population of sixteen individuals randomly sampled from a uniform distribution spanning the admissible domain and repeated in a multi-start approach. To handle comparable residuals and to ensure that the fitting protocol was not biased, we shaped the objective function as a weighted absolute distance between the predictions and the experimental data. We suitably bounded the parameter search space to guarantee biological-meaningful estimations and to ease the model calibration. To handle comparable residuals and to ensure that the fitting protocol was not biased, we shaped the objective function as a weighted absolute distance between the predictions and the experimental data. We suitably bounded the parameter search space to guarantee biological-meaningful estimations and to ease the model calibration.

The uncertainty of the parameter estimates and the model output was quantified through Monte Carlo simulations considering 1,500 pairs of (CL, Ka) randomly generated parameters (Supplementary Figures S1–S4) (Gentle, 2009). The samples were drawn from two independent lognormal distributions (Carlo et al., 2023) centered in the best estimates with a coefficient of variation of 30% as in (Lyons et al., 2013) and bounded inside the admissible space of parameters.

### Sensitivity analysis

A drug-specific local sensitivity analysis was performed on AUC, C_max_, T_max_, and PK profile of plasma and lung compartments to determine the most sensitive parameters for the mPBPK model. Body weight, cardiac output, tissue-to-plasma partition coefficients for lung, kidney, liver, gut, and spleen, rate of absorption, bioavailability, total body clearance, fraction of renal clearance, blood-to-plasma ratio, and plasma free fraction were included in the analysis. Fractional volumes and flows were excluded to not bias the physiological normalization constraint via single perturbation. The sensitivity scores were measured using the logarithmic sensitivity approach (LSA) computed with a perturbation of 1% of the reference parameter value (Wu et al., 2008; Zi, 2011). This method comes with the great advantage of being dimensionless, which allows us to easily compare model parameters with different units of measure. Mathematically, if X is the chosen metric and p is the parameter of interest, the LSA was calculated as in Equation 10:

$${PK}_{{measure}_{LSA}}\left(p\right)=\frac{\partial \log\left(X\left(t,p\right)\right)}{\partial \log\left(p\right)}=\frac{\partial X\left(t,p\right)\cdot \frac{1}{X\left(t,p\right)}}{\partial p\cdot \frac{1}{p}}=\frac{\partial X\left(t,p\right)}{\partial p}\cdot \frac{p}{X\left(t,p\right)}$$

Equation 10. The equation to compute the relative logarithmic sensitivity index for a parameter p.

where the first term in the last chain of equalities can be well approximated by the first-order finite difference (Saltelli et al., 2007; Simoni et al., 2019).

To better summarize the parameter influences on the model, we only considered the maximum sensitivity index reported for each parameter and PK measure on plasma and lung across the drugs. Model rates that are functions of the perturbed parameter have been updated accordingly along the procedure to retain all chemical and biological dependencies in the analysis.

### Computational time comparison

The model was benchmarked in terms of computational time with a reference full-body PBPK model available in Matlab SimBiology (Peters, 2008; Stader et al., 2019; MathWorks SimBiology Team, 2023). The benchmark was computed by simulating isoniazid, for which the full-PBPK physiological parameters were obtained from (MathWorks SimBiology Team, 2023) using the same physicochemical properties used in the mPBPK. The minimal and full PBPK models were simulated 10 times with a mean elapsed time of 0.0848 +/- 0.0098 and 0.2639+/- 0.0306 s, respectively, under the same *ode15s* setting and PK conditions (14 daily doses of 25 mg/kg). A comparative plot for the simulation time is available in Supplementary Figure S5. Visual predictive check ensured that the global behavior of the full-body PBPK model was retained.

## Results

### Model description

We designed a novel parsimonious physiologically based pharmacokinetics model to describe anti-TB drug ADME in the mouse. Starting from the works of (Jones and Rowland-Yeo, 2013; Stader et al., 2019; Lee et al., 2020), we iteratively lumped compartments that are marginally involved in pulmonary TB infection to streamline the model diagram. As a result of an extensive validation step, adipose, brain, bone, heart, muscles, pancreas, and skin were grouped together in a compartment called “other”, whilst interstitial spaces were embedded into corresponding tissues and organs. The lumped compartment closely resembles the clustering identified by (Yau et al., 2023) to reduce PBPK models based on rat tissue composition.

The final reduced model, whose diagram is shown in Figure 1, includes nine compartments. Eight of them are physiologically-derived, venous blood, arterial blood, lung, kidney, liver, spleen, gut, and lumped tissues, whilst one is treatment-specific to accommodate the oral route of administration. In contrast to (Ryu et al., 2022), we kept systemic bloodstreams and lungs separated to gain PK insights into the target tissue and fit the experimental data. The gut, spleen, liver, and kidney, which are pivotal or contributor tissues for absorption, distribution, and elimination phases, were explicitly modeled. First-order reactions for absorption and elimination processes and for drug exchange among compartments were found to best support the PK datasets. Mechanisms of gut-reabsorption did not lead to substantial fit improvements and thus were discarded for parsimony. Rodgers and Rowland’s method was chosen for tissue-to-plasma partition coefficient computation since comparative studies demonstrated its enhanced performances across both drug classes (>70% compounds within threefold of experimental values) and tissues (from 66.1% in the brain to 92.7% accuracy in the heart) (Jones et al., 2011; Graham et al., 2012). Under the experimental setting of plasma and lung sample collection, the proposed design satisfies the global structural identifiability criterium for the two parameters to estimate, *i.e.*, absorption and total clearance rates, mitigating one of the major drawbacks ascribed to whole-body PBPK models (Chiş et al., 2011; Läer and Khalil, 2011; Peters and Dolgos, 2019). In addition, compared to a reference full-body PBPK model (Peters, 2008; Stader et al., 2019), our ODE system can be simulated more than three times faster, which results in a substantial speed up that enables larger what-if investigations.

### Model calibration and validation

We tested our minimal PBPK model on a variety of compounds with several doses in training and validation (Frechen and Rostami-Hodjegan, 2022). The uncertainty on the outputs was estimated through Monte Carlo simulations and all the successive analyses leveraged the 1500-mice virtual population (VP) generated with this technique. The details are provided in the Methods section.

**FIGURE 2:**
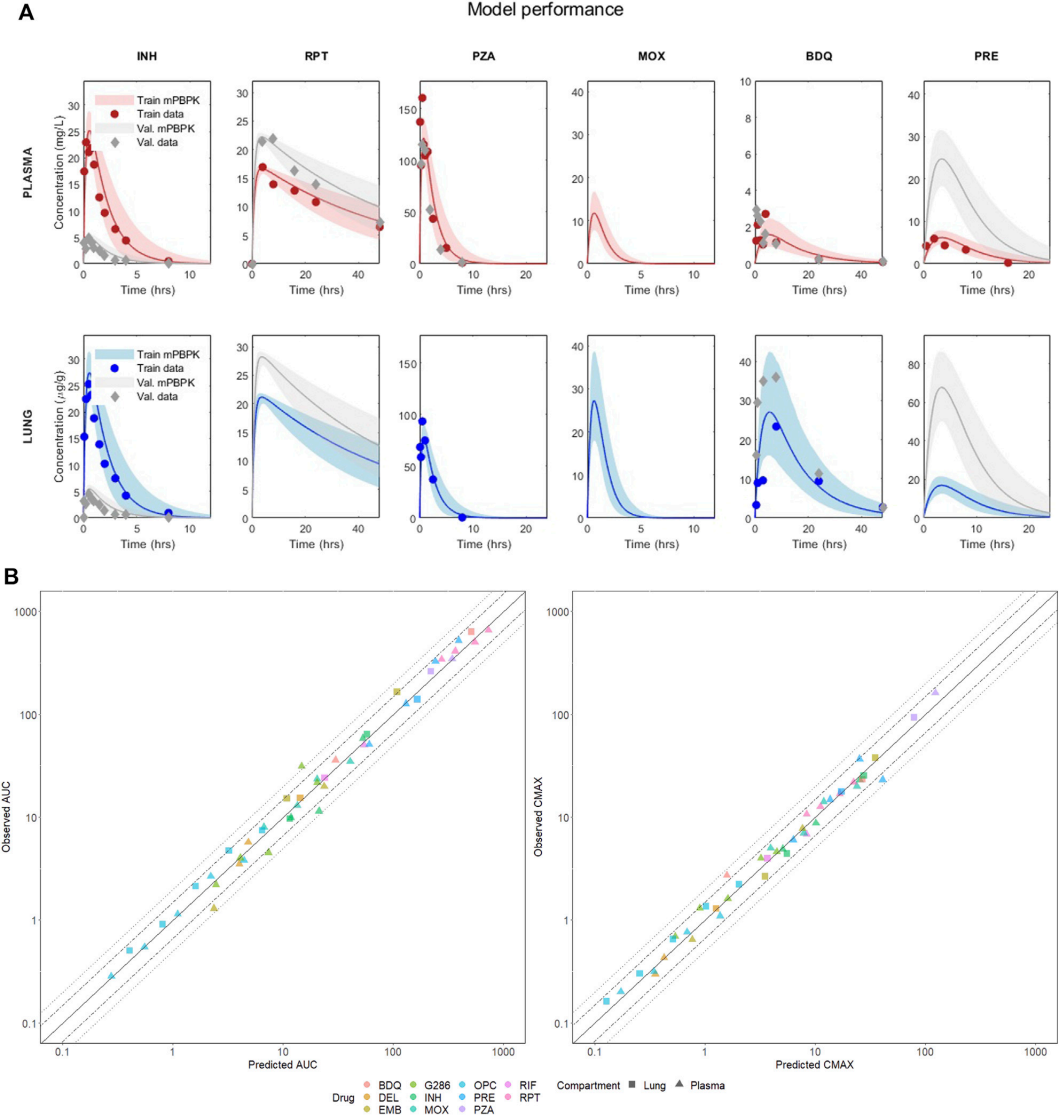
**(A)** Visual predictive check of the mPBPK model for six drugs in plasma and lung. The figure shows the performance in training and validation for the mPBPK model in plasma and lung. All figures show the median of the simulated VP (solid line) and the five and ninety-five percentiles (shaded area); dots represent the experimental data at each time point (or their mean if multiple measurements were available). Red refers to the training sets in plasma, blue to the training sets in lungs, and grey are the validation sets. INH—isoniazid (training 25 mg/kg, validation 5 mg/kg); RPT—rifapentine (training 15 mg/kg, validation 20 mg/kg); PZA—pyrazinamide (training 150 mg/kg, validation 150 mg/kg); MOX—moxifloxacin (training 100 mg/kg, validation 100 mg/kg); BDQ—bedaquiline (training 25 mg/kg, validation 25 mg/kg); PRE—pretomanid (training 25 mg/kg, validation 100 mg/kg). **(B)** Correlation plots between observed and best-fit predicted AUCs and Cmax, color-coded for drugs and shape-coded for compartments. Solid lines are the theoretical perfect agreement reference lines (bisector), while the dashed lines mark the 1.5- and two-fold from reference. Observed AUCs are computed via the trapezoidal rule.

The model’s performance for six anti-TB compounds, including isoniazid, rifapentine, pyrazinamide, moxifloxacin, bedaquiline, and pretomanid, in both training and validation is presented in Figure 2A and summarized in Table 1. All the panels display the median of the simulated VP (solid line), five and ninety-five percentiles (shaded area) and the PK observables (red dots) when literature-accessible. If no time series were available, the model was trained on AUC and Cmax static data. In parallel, Table 1 reports the corresponding quantitative description of the mentioned compounds, providing the median, five, and ninety-five percentiles of the AUC and Cmax across all training and validation doses. Comprehensive accuracy plots of the model-predicted AUC and Cmax compared to the observed data for all compounds and dosages are shown in Figure 2B. For a full visualization of the model’s performance and a summary of observed and predicted PK indexes, we refer to Supplementary Figures S1–S4 and Supplementary Table S3.

**TABLE 1:** Predicted *versus* observed AUC and Cmax in plasma and lung for the 6 drugs at the training and validation doses visualized in Figure 2.

Drug	Dose (mg/kg)	Train	Val.	Plasma AUC_0-t_ (mg*h/L)		Plasma CMAX (mg/L)		Lung AUC_0-t_ (μg*h/g)		Lung CMAX (μg/g)	
				Obs.	Pred.	Obs.	Pred.	Obs.	Pred.	Obs.	Pred.
INH	5		√	9.93	12.38 (7.27–19.58)	4.91	5.03 (4.11–5.76)	9.7	11.97 (7.63–16.76)	4.44	5.48 (4.48–6.28)
	25	√		58.56	54.93 (35.01–76.86)	22.93	25.17 (20.57–28.83)	63.89	59.87 (38.17–83.78)	25.28	27.44 (22.42–31.43)
RPT	15	√		503.7	562.38 (437.79–654.06)	16.94	16.66 (15.79–17.21)	NA	714.22 (555.99–830.65)	NA	21.16 (20.06–21.86)
	20		√	657.84	749.79 (583.83–872.07)	21.94	22.21 (21.06–22.95)	NA	952.24 (741.45–1,107.53)	NA	28.21 (26.74–29.15)
PZA	150	√	√	346.1	358.37 (211.54–557.40)	161.2	121.81 (97.25–141.88)	262.8	231.01 (136.36–359.30)	93.6	78.52 (62.69–91.46)
MOX	100	√		23.58	21.09 (11.82–36.96)	14.18	11.80 (7.79–16.74)	NA	48.73 (27.31–85.38)	NA	27.26 (18.00–38.66)
	200		√	34.84	42.19 (23.64–73.92)	20.04	23.60 (15.58–33.46)	NA	94.77 (65.16–149)	NA	54.53 (41.67–72.65)
BDQ	25	√	√	35.9	30.83 (16.31–57.08)	2.72	1.58 (0.94–2.49)	635.9	527.56 (279.06–976.65)	23.3	27.07 (16.00–42.54)
PRE	25	√		50.9	62.16 (35.63–100.17)	6	6.18 (4.5–7.87)	139.9	170.32 (97.64–274.48)	17.8	16.94 (12.53–21.57)
	100		√	327.6	248.61 (142.52–400.70)	36.81	24.74 (18.28–31.49)	NA	681.19 (390.51–1,097.93)	NA	67.78 (50.09–88.27)

Median, five and ninety-five-percentile intervals are reported. AUC_0-t_ refers to AUC_0-24_ for all drugs except INH and PZA for which an AUC_0-8_ was computed as in the data. Abbreviations: INH - isoniazid; RPT - rifapentine; PZA - pyrazinamide; MOX - moxifloxacin; BDQ - bedaquiline; PRE - pretomanid.

The minimal design can correctly reconstruct the PK dynamics, AUC, and Cmax for all the considered compounds, supporting several drug classes (including diarylquinolines, rifamycins, fluoriquinolones, nitroimidazooxazines, and carbostyril derivative) and mechanisms of action (inhibition of cell wall synthesis, fatty acids synthesis, ATP generation, DNA replication, RNA synthesis, and cholesterol catabolism) at once with comparable prediction accuracy. Aggregating both training and validation in the statistics, 100% Cmax and 97.87% AUC lay within the two-fold difference from the observed values, meeting the reference model-fidelity criterium (Wagner et al., 2015). Only the plasma AUC of GSK286 18 mg/kg, employed here for validation, exceeds the two-fold values, whereas all other doses are well captured. Moreover, 89.36% of the AUC and 95.74% of Cmax are within 1.5-fold.

**FIGURE 3:**
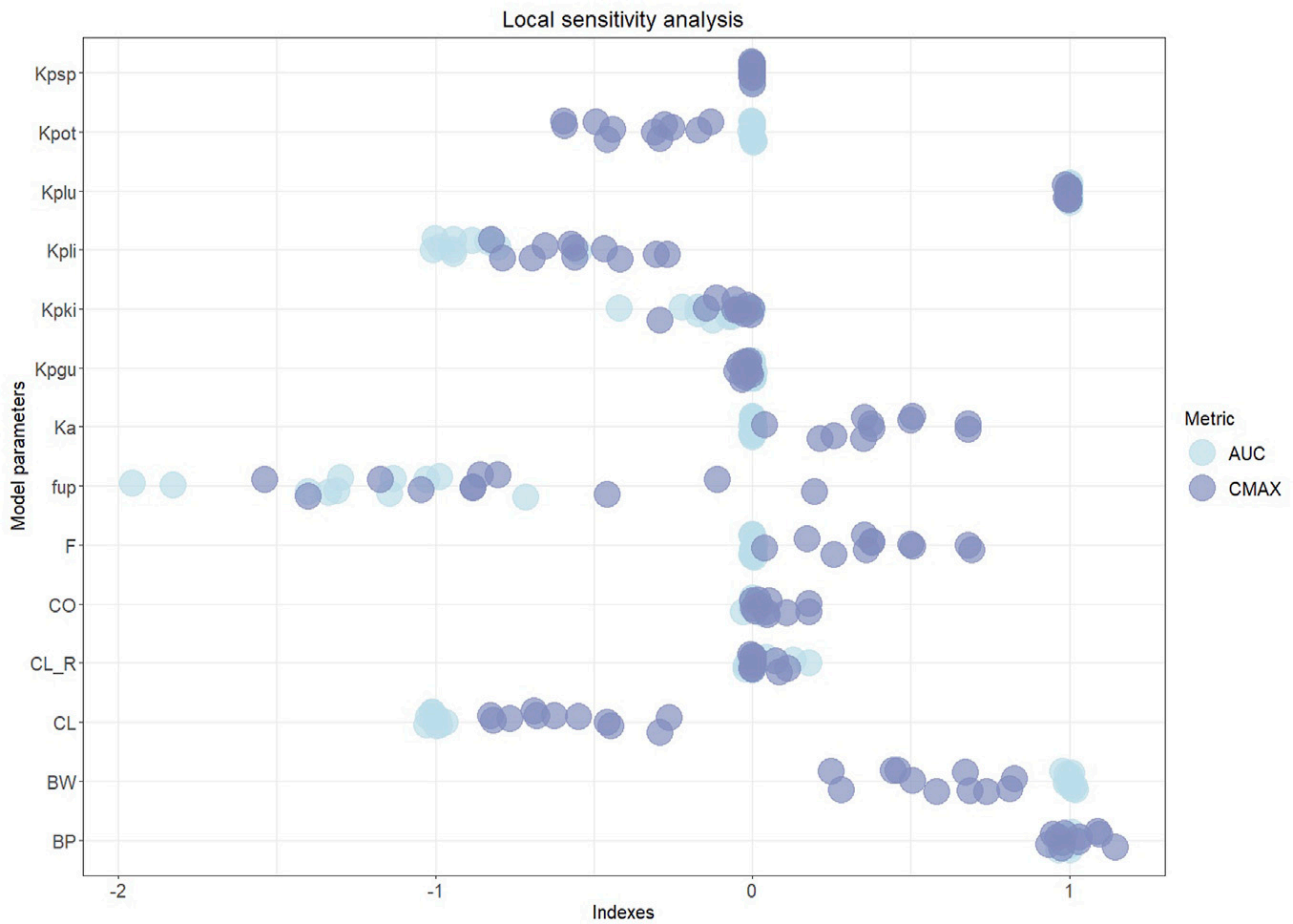
Sensitivity analysis indexes color-coded by relevant PK metrics with row-aligned jittered dots representing the 11 drugs. Drug labels are omitted to provide a unified analysis and improve the visualization.

The local sensitivity analysis we performed on the model listed AUC and Cmax as the most informative measures in terms of model response to perturbations. Tmax proved to have a quasi-null sensitivity, whilst PK profiles were well-summarized by AUC and Cmax. As shown in Figure 3, sensitivity indexes spanned the [-1.9557, 1.0264] and [-1.53757, 1.1453] intervals for AUC and Cmax, respectively, with fu_p_, BP, CL, BW, Kp_li_, Kp_lu_, F, Ka, Kp_ot_, and Kp_ki_ resulting as the overall top ten sensitive parameters. The result agrees with (Yau et al., 2020) on the pivotal role of fu_p_ and BP in mechanistically driving the drug disposition as captured by our physiological framework.

The sensitivity ranking points out the necessity of coherent literature sources and shared databases to compute tissue-to-plasma partition coefficients and to set *a priori* fixed parameters. In particular, the analysis calls for a standardized procedure in the experimental measurement of fu_p_ and BP to increase the reliability and transversality of PBPK-based models. Furthermore, the high sensitivity of the estimated parameter CL enhances the identifiability achievement of our minimal design and the consequent reliability of the results.

### Support for experimental designs

After a broad validation and structural inspection of the minimal PBPK model, we compared compound levels in the systemic circulation and in the target tissue at literature human-equivalent doses (Bartelink et al., 2017; Chen et al., 2017; Pieterman et al., 2021; Mudde et al., 2022), discussing the impact of the sampling site on predicted efficacy indexes at steady state. As depicted in Figure 2, bedaquiline exhibited the greatest penetration in the lungs, with a steady-state AUC more than 17 times higher compared to plasma, in accordance with the literature (Irwin et al., 2016; Muliaditan and Della Pasqua, 2022; Mehta et al., 2023). Ethambutol, delamanid, pretomanid, and moxifloxacin also showed increased lung exposure, although to a lesser extent, with lung-to-plasma ratios ranging from approximately 2.3–4.6 (Strydom et al., 2019; Mehta et al., 2023). OPC, G286, isoniazid, and rifapentine demonstrated comparable lung and plasma AUCs (with ratios between 1.09 and 1.46), whereas rifampicin and pyrazinamide showed a reduction of the ratio to 0.44 and 0.66, respectively, mirroring clinical results (Strydom et al., 2019). Furthermore, most of the considered drugs showed a full washout within a single dosage interval in both plasma and lung. In contrast, as depicted in Supplementary Figure S6, model-based simulations for RPT and BDQ suggested an accumulation in the target tissue that stabilizes after 2 weeks of daily administration.

Motivated by the differential plasma-to-lung partitioning at steady-state, a comprehensive compartmental evaluation of the pharmacodynamic target attainments was conducted for each drug at human equivalent doses. We analyzed four *in vitro* potency metrics associated with various bacterial growth and persistence conditions of wild-type *M. tuberculosis* (Supplementary Table S1). Namely, we considered the minimum inhibitory concentration (MIC50) to reduce bacterial growth by 50%, the minimum bactericidal concentration to kill 90% viable bacteria under aerobic, nutrient-rich conditions (MBC90), the minimum concentration required to inhibit 90% growth in macrophage (MacroIC90), and the minimum bactericidal concentration achieving a one-log kill of non-replicating bacteria under hypoxic, nutrient-rich conditions (Wayne Cidal concentration or WCC90) (Wayne and Hayes, 1996; Lakshminarayana et al., 2015). Whilst the first two metrics are aspecific, MacroIC90 and WCC90 are derived from media that mimic fibrotic and hypoxic intra-lung portions, respectively, thus representing harder-to-treat histopathological scenarios. In accordance with the literature (Strydom et al., 2019; Ernest et al., 2021; Mehta et al., 2023) the investigation has been carried out by comparing total drug levels in plasma and lungs with chosen cutoffs, leaving plasma-protein-binding correction factors out. To be noted, the 10% fetal bovine serum (FBS) added to the medium employed for MacroIC90 intrinsically accounts for protein binding limiting possible binding-related biases (Robertson et al., 2021).

Table 2 collects the first, second, and third quartiles of the simulated plasma and target tissue coverage at steady-state, i.e., time above metrics, for the mice virtual population, while Supplementary Figure S7 provides the corresponding full visualization. As shown in Table 2, for 4 compounds—rifapentine, delamanid, OPC, and G286—the efficacious time in plasma was a good indicator for lungs, with 95% virtual population having relative discrepancies between the two compartments smaller than 10% for all available sterilizing and bactericidal metrics. Pretomanid, isoniazid, and rifampicin showed a similar trend under all bacterial conditions except WCC90, which resulted in around 48%, 12%, and −25% relative change of the lung efficacious time against the systemic circulation as reported in Table 2.

**TABLE 2:** Target attainment in plasma and lungs of the considered compounds at human equivalent doses in terms of hours and relative change between plasma and lungs.

	Comp.	RPT	RIF	BDQ	DEL	PRE	INH	OPC	G286 (*)	PZA	MOX	EMB
MIC50	Plasma	24 (24–24)	24 (24–24)	24 (24–24)	24 (24–24)	24 (21.7–24)	11.5 (7.2–18.3)	11.6 (8.1–17.2)	10.2 (7.2–14.3)	5.7 (3.7–9)	4.9 (3.6–6.9)	5.5 (3.8–7.9)
	Lung	24 (24–24)	24 (24–24)	24 (24–24)	24 (24–24)	24 (24–24)	11.6 (7.3–18.6)	12.4 (8.6–18.4)	10.6 (7.5–15)	4.8 (3.1–7.6)	5.6 (4.1–7.8)	8.2 (6–11.8)
	∆ (%)	0	0	0	0	0	0.87	6.9	3.92	−15.79	14.29	49.09
MBC90	Plasma	24 (24–24)	24 (24–24)	0 (0–0)	24 (24–24)	24 (21.3–24)	11.3 (7.1–18.1)	11.6 (8.1–17.2)	NA	6.2 (4–9.8)	4.6 (3.4–6.4)	6 (4.2–8.6)
	Lung	24 (24–24)	24 (22.9–24)	24 (21.8–24)	24 (24–24)	24 (24–24)	11.5 (7.2–18.3)	12.4 (8.6–18.4)	NA	5.3 (3.4–8.4)	5.3 (3.9–7.4)	8.7 (6.3–12.5)
	∆(%)	0	0	inf	0	0	1.77	6.9	NA	−14.52	15.22	45
MacroIC90	Plasma	24 (24–24)	24 (20.3–24)	24 (21–24)	24 (22.4–24)	24 (19–24)	12.2 (7.6–19.5)	17.4 (11.8–24)	NA	9 (5.8–14.3)	3.2 (2.4–4.6)	2.7 (1.3–4.2)
	Lung	24 (24–24)	23.6 (16.8–24)	24 (24–24)	24 (24–24)	24 (22.4–24)	12.3 (7.7–19.7)	18.1 (12.3–24)	NA	8.2 (5.2–12.9)	3.9 (2.9–5.5)	5.7 (4–8.2)
	∆(%)	0	−1.66	0	0	0	0.82	4.02	NA	−8.89	21.88	111.11
WCC90	Plasma	24 (24–24)	22.8 (15.3–24)	0 (0–5.60)	NA	12.6 (8.3–19.4)	1.7 (0.9–2.9)	NA	NA	5.8 (3.7–9.1)	1.9 (1.3–2.9)	0 (0–0)
	Lung	24 (24–24)	17.10 (11.2–24)	24 (24–24)	NA	18.4 (12.6–24)	1.9 (1.1–3.1)	NA	NA	4.9 (31–7.7)	2.8 (2–3.9)	2.4 (1.1–3.8)
	∆ (%)	0	−25	inf	NA	47.62	11.76	NA	NA	−15.52	47.37	inf

The table shows the median time in which the compounds are above the PD metrics and the corresponding five and ninety-five percentiles values computed from the virtual population. NA: not available; inf: result of the relative error formula when the baseline is 0. For GSK-2556286, only MIC90 was available and has been used in place of the MIC50 for this analysis. Abbreviations: RPT—rifapentine; RIF—rifampicin; BDQ—bedaquiline; DEL—delamanid; PRE—pretomanid; INH—isoniazid; OPC - OPC-167832; G286 - GSK2556286; PZA—pyrazinamide; MOX—moxifloxacin; EMB—ethambutol; MIC50 - minimum inhibitory concentration to reduce bacterial growth by 50%; MBC90 - the minimum bactericidal concentration to kill 90% viable bacteria under aerobic, nutrient-rich conditions; MacroIC90 - the minimum concentration required to inhibit 90% growth in macrophage; WCC90 - minimum bactericidal concentration achieving a one-log kill of non-replicating bacteria under hypoxic, nutrient-rich conditions; Comp—compartment.

Time above targets was consistently higher at the disease-site-of-action compared to plasma for moxifloxacin and ethambutol, with an increase between 14%-48% and 45%–112%, respectively, across the metrics. Notably, ethambutol did not exceed the WCC90 concentration in plasma, but it did for 2.4 h on average in lungs. In this sense, the most remarked mismatches, with null efficacious hours in plasma and 24 in lungs, characterized PK/PD indexes of bedaquiline, which would partner the hypothesis of a TB-specific lack of correlation between plasma PK and efficacy scores as claimed in (Dartois, 2014). Together with rifampicin, pyrazinamide was the only other compound with longer efficacious intervals in the systemic circulation rather than at the target tissue (relative change from ∼8% to ∼16%).

Overall, bedaquiline, rifapentine, rifampicin, delamanid, and pretomanid showed a longer efficacious time than the other compounds, achieving at least 17 h of coverage at the site of action for all potency metrics (Table 2). In contrast, moxifloxacin, pyrazinamide, and ethambutol had the worst performances, with a maximum of ∼5.5, 8, and 9 h only above pharmacodynamic targets, respectively. Isoniazid and OPC demonstrated 11 h of effectiveness in lungs on average, with some exceptions depending on the potency metric used. For instance, isoniazid was effective above the WCC90 threshold for less than 2 h on average, while OPC maintained effectiveness above the MacroIC90 threshold for two-thirds of the entire day. G286, for which only MIC90 was available, resulted above this value for more than 10 h both in plasma and lung.

**FIGURE 4:**
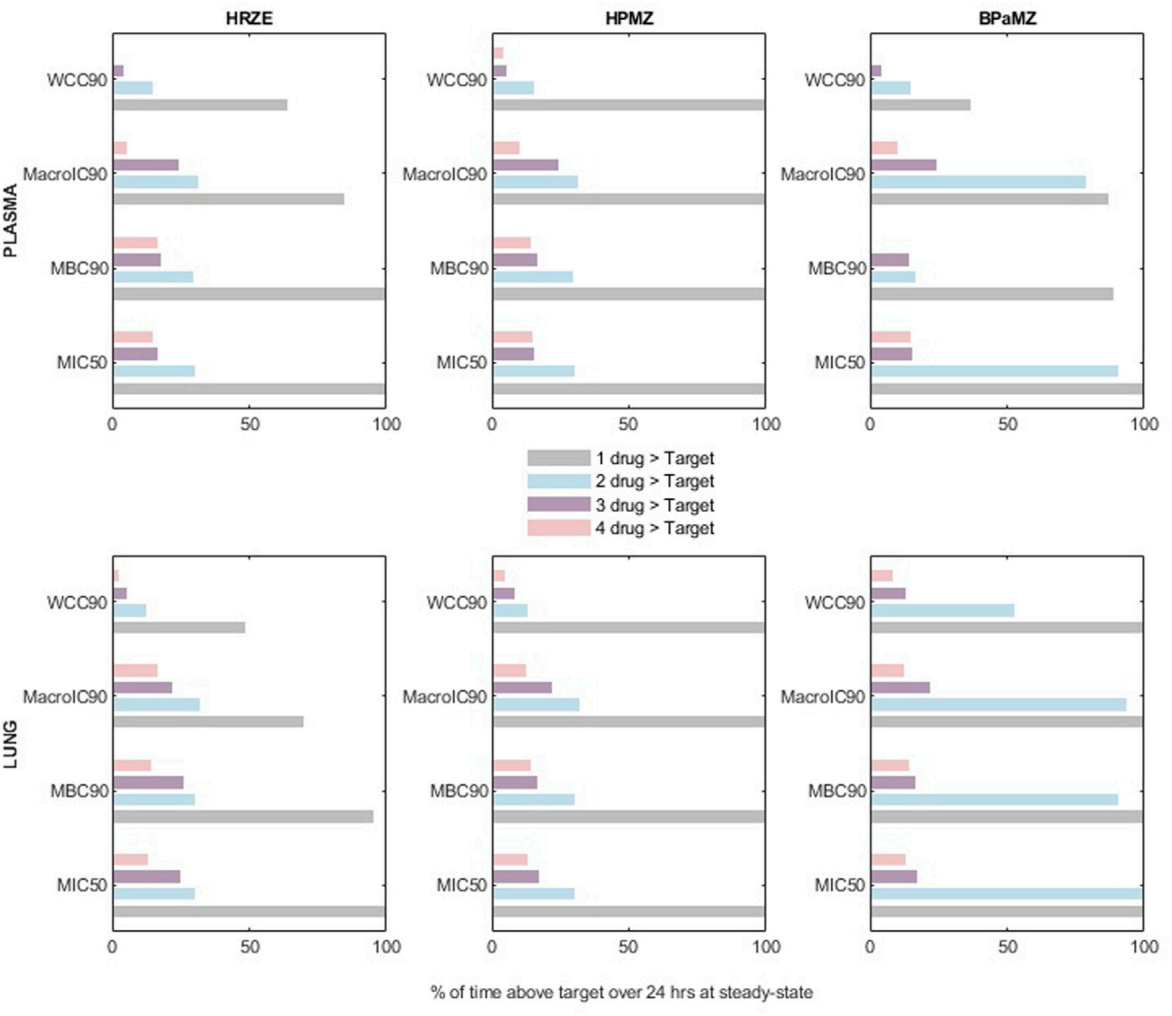
Number of compounds and percentage of time above the different efficacy targets in plasma and lungs for the regimens HRZE, BPaMZ, and HPMZ. MIC50 stands for the minimum inhibitory concentration to reduce bacterial growth by 50%, while MBC90, MacroIC90, and WCC90 refer to the minimum bactericidal concentration to kill 90% viable bacteria under aerobic, nutrient-rich conditions, within the macrophage, and under hypoxic, nutrient-rich condition, respectively.

By extension, we adapted previous pharmacodynamic analysis to drug regimens, which is the common therapeutic setting for TB. Although the model does directly account for drug-drug interactions (DDIs) since data were retrieved from single agent experiments, the use of a virtual population with up to 30% variation in absorption and clearance allows for covering the effects of mild-to-moderate DDI in the dynamics and mitigating possible biases. We examined the HRZE regimen, which is the gold standard treatment, and two promising cocktails, BPaMZ and HPMZ, recently tested in the SimpliciTB and Study 31/A5349 multicenter phase 3 trials (Salinger et al., 2019; Tweed et al., 2019; Dorman et al., 2021). We considered the reference protocol of simultaneous drug administration routinely used in clinics and we worked under the stringent setting with a drug being above the target at a certain time if 95% of its VP fulfills the condition. Figure 4 compares the number of compounds and the percentage of the dosing interval time in which the three regimens reach the pharmacodynamic cutoffs at steady-state. Consistently, the target attainment at the disease site-of-action was found to be higher for both HPMZ and BPaMZ than for HRZE, due to the superior performance of rifapentine, bedaquiline, and pretomanid. With a 100% coverage scored in lungs, both regimens ensure one compound to always reach the critical concentration under all bacterial conditions, while for HRZE this percentage can drop to around 50%. Apart from the WCC90 assay, against which the target attainment of two compounds simultaneously is guaranteed for around 55% of the day only, BPaMZ overall contributes with two compounds being effective the entire dosing interval, standing out with respect to the one-third day signed by HPMZ and HRZE. Notably, within the most hard-to-treat scenario mimicked here by the WCC90 metric, BPaMZ and HPMZ outperformed the standard of care regimen with an around two-fold longer time interval counting all regimen compounds above the efficacy threshold.

Overall, these preclinical results suggest that the latest regimens are likely to achieve faster bacterial burden reduction through a longer effective time at the site-of-action, confirming the clinical evidence.

## Discussion

Recently, minimal-PBPK models have made an impact in pharmacometrics as powerful tools to bridge top-down and bottom-up approaches (Cao and Jusko, 2012; Mavroudis et al., 2019; Bloomingdale et al., 2021; Mehta et al., 2023). By retaining essential compartments for absorption, metabolism, and clearance, our mPBPK has demonstrated its capability to accurately predict the PK dynamics of eleven anti-TB compounds with diverse MOAs and across several drug classes, regardless of their chemical characteristics or development stage. Acknowledging the significance of target-centric approaches, the model offers valuable insights into the quantitative aspects of lung PK in mice, which are proven to be crucial for optimizing dose-finding and target attainment studies. As there can be significant differences between plasma and lung levels for certain drugs, pulmonary-based experimental protocols could enhance drug exposure at the site of action, preventing resistance mechanisms by targeting different bacterial subpopulations simultaneously (Ernest et al., 2021; Landersdorfer and Nation, 2021; Allué-Guardia et al., 2022; Chesov et al., 2022; Mehta et al., 2023). In this regard, the considered four *in vitro* potencies, namely MIC50, MBC90, MacroIC90, and WCC90, allowed a comprehensive investigation of the pharmacodynamic properties of each compound against both replicating and persistent bacteria to cover standard and hard to treat TBrelated infections. (Lakshminarayana et al., 2015).

At human-equivalent doses, bedaquiline, rifapentine, delamanid, pretomanid, and rifampicin showed the top 5 coverage at the site-of-action, with most metrics met for almost the entire day. These results were well-recapitulated when drugs were simulated within regimens, with HPMZ and BPaMZ combinations displaying higher combined target attainment compared to reference HRZE. These findings, in line with clinical results, suggest that these Phase 3 regimens can achieve a longer effective time for more compounds, providing better PD indexes against different bacteria populations. In terms of regimen experimental protocols, model-based simulations indicate that compounds with a long half-life in the lungs, such as bedaquiline, rifapentine, or pretomanid, ensure adequate coverage along the whole therapeutic window and may allow the staggered administration of remaining components. Such a staggered approach could be implemented to reduce the risk of drug-drug interactions (DDIs) or, in large-scale pre-clinical settings, to allow scientists to schedule experiments more effectively or conveniently. Overall, these results advocate for the use of mechanistic and semi-mechanistic PK models, like PBPK or our mPBPK, that can quantify the achieved exposure in the site-of-action. These models may contribute to better tuning novel regimens improving their design and effectiveness or for dose optimization to achieve optimized coverage of efficacious windows (Ernest et al., 2021; Muliaditan et al., 2022; Mehta et al., 2023).

Our model can provide novel quantitative perspectives of the disposition of anti-TB drugs in mice. However, certain limitations need to be addressed. Firstly, the model currently lacks a detailed description of intra-pulmonary TB lesions, since most available datasets in the literature are based on BALB/c mice, which do not develop TB granulomas. As a result, the model was designed to support drug disposition up to the lung compartment to maintain consistency across the analysis. To improve the model, we plan to incorporate a parsimonious description of drug diffusion into TB pulmonary cellular lesions and caseum, utilizing promising C3HeB/FeJ mice (Bouté et al., 2017) or New Zealand White rabbit datasets to validate the updated design. Indeed, the model would benefit of more standardized data sources including only infected animals.

Secondly, the model could be enhanced to consider enzyme-mediated reactions to account for drug-to-drug interactions, which are known to affect the PK profiles of certain compounds when administered together. The inclusion of enzyme-dependent reactions would also improve the target attainment analysis and increase the model performance on drugs that may scale non-linearly at higher doses. However, this would deviate from the minimal design we are interested in and would significantly increase the number of parameters.

Thirdly, the model could be extended to include the description of active metabolites, which may contribute to capturing multi-phasic clearances and may be relevant for efficacy investigation. Within the analyzed drug set, only the N-monodesmethyl metabolite (M2) of BDQ would be worth of specific modeling efforts as it is active and abundant, while the efficacy of other catalyzed products is already implicitly recapitulated in the potency metrics of the corresponding parental drugs. Here, we partnered with previous literature by choosing a unique minimal model diagram that can reconstruct all the PK profiles of the included parental drugs only, leaving the inclusion of M2 metabolite for future model extension and refinement.

Last, the model translational capabilities are limited to one species only. Although mice have been workhorses in TB preclinical and translational research, accounting for other preclinical organisms, such as rabbits, could be beneficial to empower the predictability of the platform. In this regard, leveraging the present results, we first plan to develop a rabbit-dedicated version of the mPBPK model to draw model scalability relationships and assess species dependencies. Derived information will be then employed to upgrade PK predictions to humans, delivering a final clinical rendering of the mPBPK framework and a full interactive translational tool.

Despite these limitations, which may guide further developments and refinements, our all-in-one model delivers a convenient tool for comparing multiple compounds, promoting a system view in terms of tissue penetration and efficacy. The model requires a limited amount of prior knowledge and has a faster simulation time compared to full-body PBPK models, making it suitable for supporting large-scale what-if analysis, pre-clinical scenarios simulations, and clinical-oriented optimization queries on anti-TB compounds and regimens. It provides a flexible tool for model-informed drug design pipelines to speed up the development of novel anti-tuberculosis agents from bench to clinic.

## Data Availability

Publicly available datasets were analyzed in this study. This data can be found here: all references are provided in Supplementary Table S2.
