# Supplementary material for: A minimal PBPK model to accelerate preclinical development of drugs against tuberculosis
Source: Front Pharmacol. 2024 Jan 4;14:1272091. doi: 10.3389/fphar.2023.1272091 (PMC10794428; doi:10.3389/fphar.2023.1272091)
Supplement: Supplementary file 1 [file DataSheet1.PDF]

Supplementary Material

A minimal PBPK model to support and speed up the preclinical development of drugs against tuberculosis.

Authors: Federico Reali (1,*, $), Anna Fochesato (1,2,*), Chanchala Kaddi (3,+), Roberto Visintainer (1), Shayne Watson (3), Micha Levi (3), Véronique Dartois (4), Karim Azer (3,§), and Luca Marchetti (1,5,$)

Institution: (1) Fondazione The Microsoft Research – University of Trento Centre for Computational and Systems Biology (COSBI), Italy; (2) University of Trento, Department of Mathematics, Italy; (3) Gates Medical Research Institute, USA; (4) Hackensack Meridian Health, USA; (5) University of Trento, Department of Cellular, Computational and Integrative Biology (CIBIO), Italy.

*: equal contribution

+: current affiliation: Sanofi, Cambridge, USA

§: current affiliation: Axcella Health, Cambridge, USA

$: corresponding authors

Supplementary Tables

Drug	Acronym	Consensus LogP	pKa	Free fraction in plasma (fup)	Blood: plasma ratio (BP)	Bio - availability (%)	Fraction of renal clearance (%)	MIC50 (mg/L)	MBC90 (mg/L)	MacroIC90 (mg/L)	WCC90 (mg/L)
Rifampicin	RIF	4.01	1.70, 7.90	0.029	0.9	1	19	0.02	0.06	0.26	0.82
Pyrazinamide	PZA	-0.95	0.87, 13.90	0.59	0.79	0.8	11.50	12.5	9.85	2.46	12.31
Bedaquiline	BDQ	6.25	9.1	0.0005	1	0.56	0	0.03	5.56	0.35	2.78
Delamanid	DEL	3.79	-	0.003	0.73	0.91	5	0.002	0.003	0.01	-
Moxifloxacin	MOX	0.83	6.25, 9.29	0.606	1.1	0.9	25	0.125	0.185	1	4.01
OPC167832	OPC	3.24	1.5	0.12	0.7	0.9	0	0.002	0.002	0.0001	-
GSK-2556286	G286	1.61	4	0.14	1	0.99	50	0.3953 (*)	-	-	-
Ethambutol	EMB	0.06	6.5, 9.55	0.83	1.3	0.57	5.5	1	0.765	4.09	20.43
Isoniazid	INH	-0.7	1.82	0.95	0.825	1	15.5	0.06	0.065	0.04	13.71
Pretomanid	PTM	2.75	7	0.136	1.72	1	1	0.2	0.23	0.45	7.19
Rifapentine	RPT	4	7.01, 7.98	0.02	1	0.7	17	0.01	0.07	0.03	0.44

Supplementary Table 1: physicochemical and pharmacodynamical properties of the considered eleven compounds. All the properties are retrieved from the literature: (Gaohua et al.*, 2015; Lakshminarayana* et al.*, 2015; Shimokawa* et al.*, 2015; Wallace, Philley and Griffith, 2015; Kaniga* et al.*, 2016; Yu* et al.*, 2016; Alfarisi* et al.*, 2017; Lyons, 2018; Strydom* et al.*, 2019; Bigelow* et al.*, 2021; Humphries* et al.*, 2021; Robertson* et al.*, 2021; Shibata* et al.*, 2021; Litjens* et al.*, 2022; Mudde* et al.*, 2022; Muliaditan* et al.*, 2022). *: for GSK-2556286 only MIC90 was available and has been used in place of MIC50 for the analysis.*

Drug	Dose (mg/kg)	Training	Source	Validation	Source
Rifampicin	10	√	(Muliaditan and Della Pasqua,2022)	√	(Bruzzese *et al.*, 2000; Rosenthal *et al.*, 2007)
Pyrazinamide	150	√	(Irwin *et al.*, 2016)	√	(Muliaditan and Della Pasqua, 2022)
Bedaquiline	25	√	(Irwin *et al.*, 2016)	√	(Irwin *et al.*, 2016)
Delamanid	2.5	√	(Hirao *et al.*, 2015)	√	(Sasahara *et al.*, 2015)
Moxifloxacin	33			√	(Poissy et al, 2010)
	66			√	(Poissy et al, 2010)
	100	√	(Rosenthal *et al.*, 2005)		
	200	√	(Rosenthal *et al.*, 2005)		
OPC167832	0.625			√	(Hariguchi *et al.*, 2020)
	1.25	√	(Hariguchi *et al.*, 2020)		
	2.5	√	(Hariguchi *et al.*, 2020)		
	5			√	(Hariguchi *et al.*, 2020)
	10	√	(Hariguchi *et al.*, 2020)		
GSK-2556286	3	√	(Nuermberger *et al.*, 2022)		
	5			√	(Nuermberger *et al.*, 2022)
	9	√	(Nuermberger *et al.*, 2022)		
	18			√	(Nuermberger *et al.*, 2022)
	25	√	(Nuermberger *et al.*, 2022)		
Ethambutol	10			√	(Muliaditan and Della Pasqua, 2022)
	100	√	(Muliaditan and Della Pasqua, 2022)		
Isoniazid	5			√	(Muliaditan and Della Pasqua, 2022)
	10			√	(Jayaram *et al.*, 2004)
	25	√	(Muliaditan and Della Pasqua, 2022)		
Pretomanid	25	√	(Lakshminarayana *et al.*, 2014)		
	54	√	(Ahmad *et al.*, 2011)		
	100			√	(Nuermberger *et al.*, 2006)
	162			√	(Ahmad *et al.*, 2011)
Rifapentine	7.5			√	(Rosenthal *et al.*, 2007)
	10	√	(Rosenthal *et al.*, 2007)		
	15	√	(Rosenthal *et al.*, 2007)		
	20			√	(Rosenthal *et al.*, 2007)

Supplementary Table 2: Reference to the literature data sources for training and validation datasets.

Drug	Acronym	Rate of absorption Ka (1/h)	Total clearance CL (L/h)	Lung-to-plasma partition coefficient (Kplu)
Rifampicin	RIF	0.3713	0.037	0.441
Pyrazinamide	PZA	3.606	0.021	0.644
Bedaquiline	BDQ	0.142	29.190	17.11
Delamanid	DEL	0.192	3.342	2.975
Moxifloxacin	MOX	1.908	0.058	2.31*
OPC167832	OPC	0.603	0.405	1.49
GSK-2556286	G286	0.387	0.186	1.184**
Ethambutol	EMB	2.472	0.009	4.60
Isoniazid	INH	4.158	0.014	1.09
Pretomanid	PTM	0.420	0.044	2.74
Rifapentine	RPT	1.674	0.019	1.27*

*Supplementary Table 3: Best fitted parameter estimates for the rate of absorption, total clearance and in vivo adjusted lung-to-plasma partition coefficient for all the compounds. * are the interspecies lung partition coefficient from literature. ** RR predicted lung partition coefficient.*

Drug	Dose (mg/kg)	Plasma AUC (mg*h/L)		Plasma CMAX (mg/L)		Lung AUC (ug*h/g)		Lung CMAX (ug/g)	
		Obs.	Pred.	Obs.	Pred.	Obs.	Pred.	Obs.	Pred.
RIF	10	50.25	53.34 (37.72-69.96)	6.82	8.17 (6.16-10.26)	24.24	23.52 (16.64-30.85)	3.98	3.60 (2.71-4.53)
PZA	150	346.1	358.37 (211.54-557.40)	161.2	121.81 (97.25-141.88)	262.8	231.01 (136.36-359.30)	93.6	78.52 (62.69-91.46)
BDQ	25	35.9	30.83 (16.31-57.08)	2.72	1.58 (0.94-2.49)	635.9	527.56 (279.06-976.65)	23.3	27.07 (16.00-42.54)
DEL	2.5	3.51	4.11 (2.28-6.86)	0.3	0.35 (0.23-0.51)	NA	12.24 (6.78-20.39)	NA	1.04 (0.67-1.51)
	3	5.7	4.94 (2.74-8.23)	0.43	0.42 (0.27-0.61)	15.39	14.69 (8.14-24.48)	1.29	1.25 (0.81-1.81)
MOX	33	8	6.96 (3.90-12.20)	5	3.89 (2.57-5.52)	NA	16.08 (9.01-28.18)	NA	9 (5.94-12.76)
	66	13	13.92 (7.80-24.39)	7	7.79 (5.14-11.05)	NA	32.17 (18.03-56.35)	NA	17.99 (11.88-25.52)
	100	23.58	21.09 (11.82-36.96)	14.18	11.80 (7.79-16.74)	NA	48.73 (27.31-85.38)	NA	27.26 (18.00-38.66)
	200	34.84	42.19 (23.64-73.92)	20.04	23.60 (15.58-33.46)	NA	97.46 (54.61-170.77)	NA	54.52 (35.99-77.30)
OPC	0.625	0.28	0.28 (0.15-0.51)	0.09	0.08 (0.05-0.14)	0.51	0.41 (0.22-0.72)	0.16	0.12 (0.08-0.20)
	1.25	0.54	0.56 (0.30-1.02)	0.2	0.17 (0.10-0.27)	0.91	0.81 (0.44-1.44)	0.3	0.25 (0.15-0.40)
	2.5	1.14	1.12 (0.59-2.03)	0.31	0.33 (0.20-0.54)	2.13	1.63 (0.88-2.88)	0.65	0.50 (0.31-0.81)
	5	2.65	2.24 (1.19-4.06)	0.76	0.67 (0.41-1.08)	4.74	3.26 (1.76-5.77)	1.36	0.99 (0.61-1.61)
	10	3.79	4.47 (2.38-8.13)	1.09	1.33 (0.82-2.16)	7.41	6.52 (3.52-11.53)	2.22	1.99 (1.22-3.22)
G286	3	2.21	2.52 (1.31-4.68)	0.69	0.53 (0.32-0.81)	NA	2.98 (1.56-5.54)	NA	0.63 (0.38-0.96)
	5	4.01	4.20 (2.19-7.79)	1.3	0.88 (0.53-1.36)	NA	4.97 (2.59-9.23)	NA	1.05 (0.63-1.61)
	9	4.51	7.56 (3.94-14.03)	1.61	1.59 (0.96-2.44)	NA	8.95 (4.67-16.62)	NA	1.89 (1.13-2.89)
	18	31.32	15.12 (7.88-28.05)	3.97	3.19 (1.91-4.88)	NA	17.90 (9.34-33.22)	NA	3.77 (2.27-5.78)
	25	21.64	20.99 (10.95-38.96)	4.6	4.42 (2.66-6.78)	NA	24.87 (12.97-46.14)	NA	5.24 (3.15-8.03)
EMB	10	1.29	2.48 (1.43-4.02)	0.64	0.76 (0.54-1.01)	15.2	11.39 (6.59-18.48)	2.66	3.51 (2.48-4.65)
	100	19.87	24.75 (14.32-40.16)	7.77	7.63 (5.38-10.12)	165.49	113.87 (65.89-184.73)	37.82	35.11 (24.76-46.54)
INH	5	9.93	12.38 (7.27-19.58)	4.91	5.03 (4.11-5.76)	9.7	11.97 (7.63-16.76)	4.44	5.49 (4.48-6.28)
	10	11.45	21.98 (14.01-30.75)	8.71	10.07 (8.23-11.53)	NA	23.95 (15.27-33.51)	NA	10.97 (8.97-12.57)
	25	58.56	54.93(35.01-76.87)	22.93	25.17 (20.57-28.83)	63.89	59.87 (38.17-83.78)	25.28	27.44 (22.42-31.43)
PRE	25	50.9	62.16 (35.63-100.17)	6	6.18 (4.57-7.87)	139.9	170.32 (97.64-274.48)	17.8	16.94 (12.53-21.57)
	54	126.63	134.38 (76.96-216.40)	14.88	13.36 (9.87-17.00)	NA	369.71 (210.87-592.93)	NA	36.60 (27.05-46.59)
	100	327.6	248.61 (142.52-400.70)	36.81	24.74 (18.28-31.49)	NA	681.19 (390.51-1097.93)	NA	67.78 (50.09-86.27)
	162	522.83	402.78(230.94-649.26)	23.09	40.07 (29.63-51.03)	NA	1103.61 (632.77-1778.97)	NA	109.78 (81.18-139.82)
RPT	7.5	342.79	281.18 (218.90-327.02)	10.67	8.33 (7.90-8.61)	NA	357.09 (278.00-415.31)	NA	10.58 (10.03-10.93)
	10	411.27	374.89(219.89-436.02)	12.69	11.11 (10.53-11.48)	NA	476.11 (370.70-553.74)	NA	14.10 (13.37-14.57)
	15	503.7	562.38(437.79-654.06)	16.94	16.66 (15.79-17.21)	NA	714.22 (555.99-830.65)	NA	21.16 (20.06-21.86)
	20	657.84	749.79(583.82-872.07)	21.94	22.21 (21.06-22.95)	NA	952.24 (741.45-1107.53)	NA	28.21 (26.74-29.15)

Supplementary Table 4: Predicted vs observed AUC and Cmax for all the compounds and all the doses. Median, 5%, and 95% quantiles intervals are reported.

Supplementary Figures

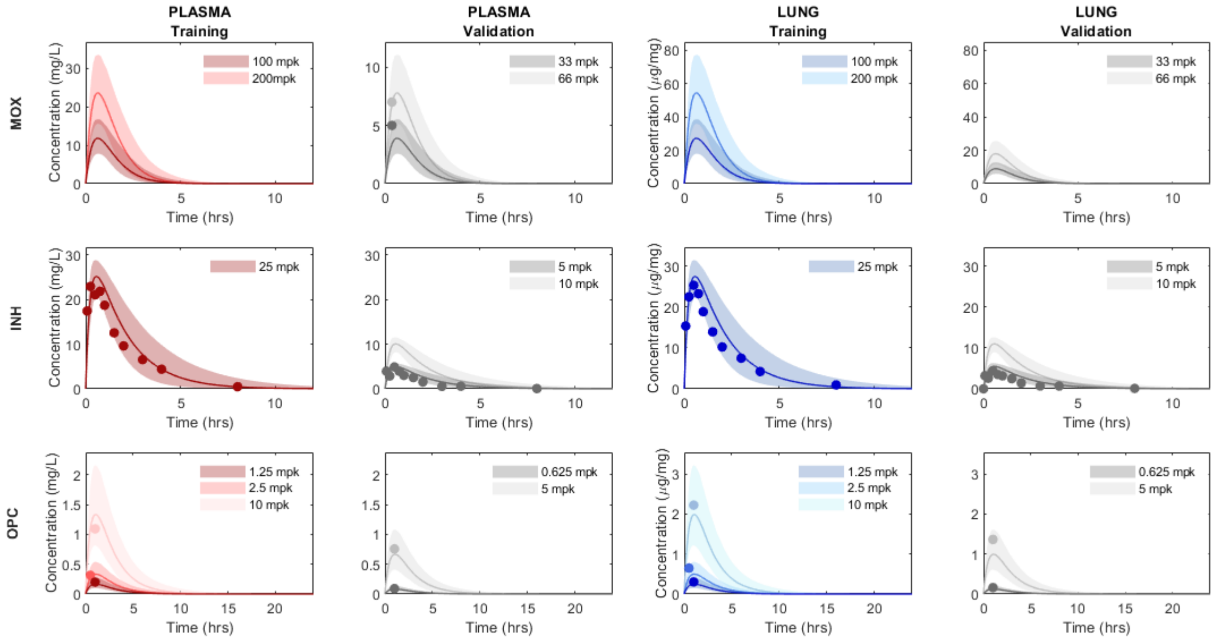

Supplementary Figure 1: Visual predictive check of the mPBPK model for moxifloxacin (MOX), isoniazid (INH), and OPC-167832 (OPC) in plasma and lung. The figure shows the performance in training and validations for the mPBPK model in plasma and lung for all the doses. All figures show the median of the simulated VP (solid line) and the fifth and ninety-fifth quantiles (shaded area). Dots represent the experimental data. Red refers to the training sets in plasma, blue to the training sets in lungs, and grey are the validation sets.

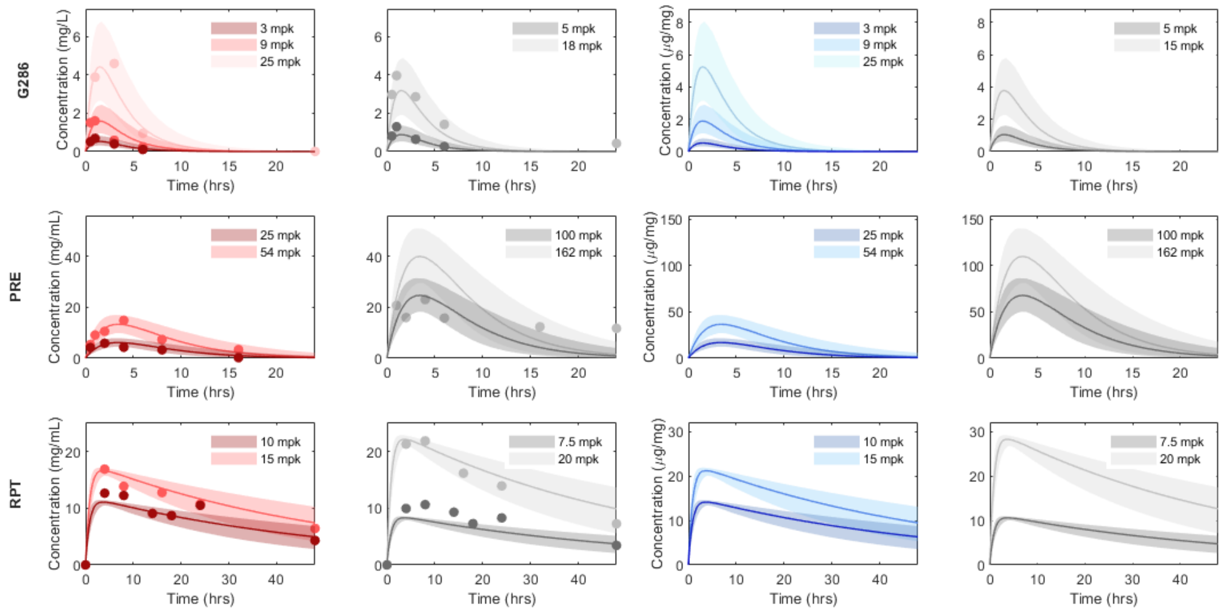

Supplementary Figure 2: Visual predictive check of the mPBPK model for GSK255286 (G286), pretomanid (PRE), and rifapentine (RPT) in plasma and lung. The figure shows the performance in training and validations for the mPBPK model in plasma and lung for all the doses. All figures show the median of the simulated VP (solid line) and the fifth and ninety-fifth quantiles (shaded

area). Dots represent the experimental data. Red refers to the training sets in plasma, blue to the training sets in lungs, and grey are the validation sets.

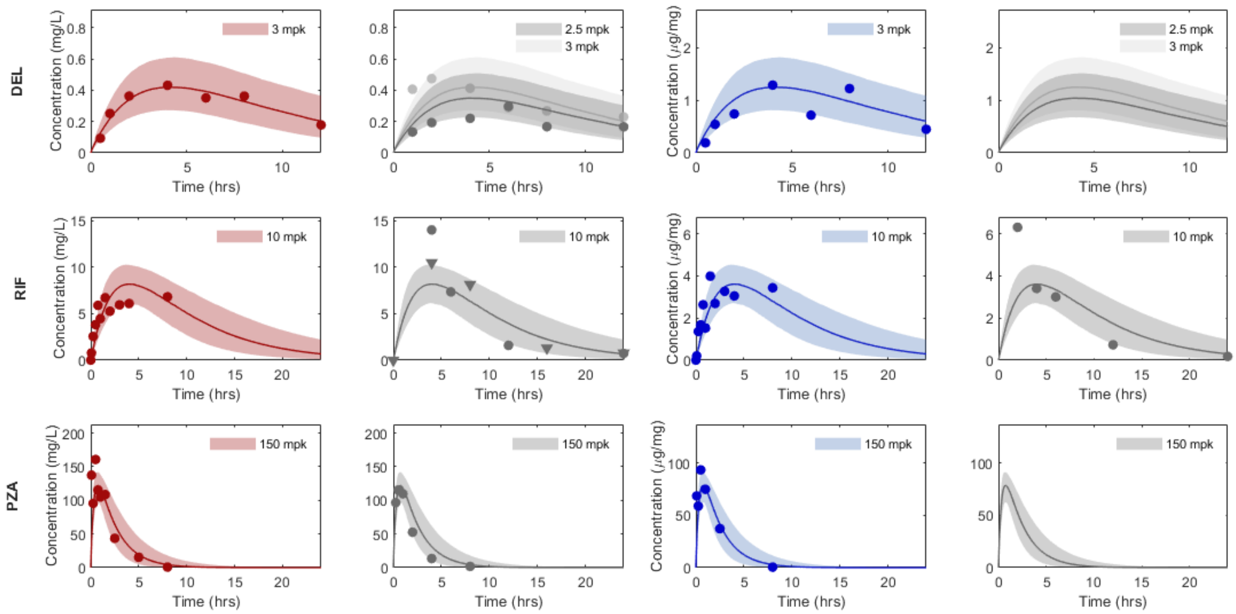

Supplementary Figure 3: Visual predictive check of the mPBPK model for delamanid (DEL), rifampicin (RIF), and pyrazinamide (PZA) in plasma and lung. The figure shows the performance in training and validations for the mPBPK model in plasma and lung for all the doses. All figures show the median of the simulated VP (solid line) and the fifth and ninety-fifth quantiles (shaded area). Dots represent the experimental data. Red refers to the training sets in plasma, blue to the training sets in lungs, and grey are the validation sets.

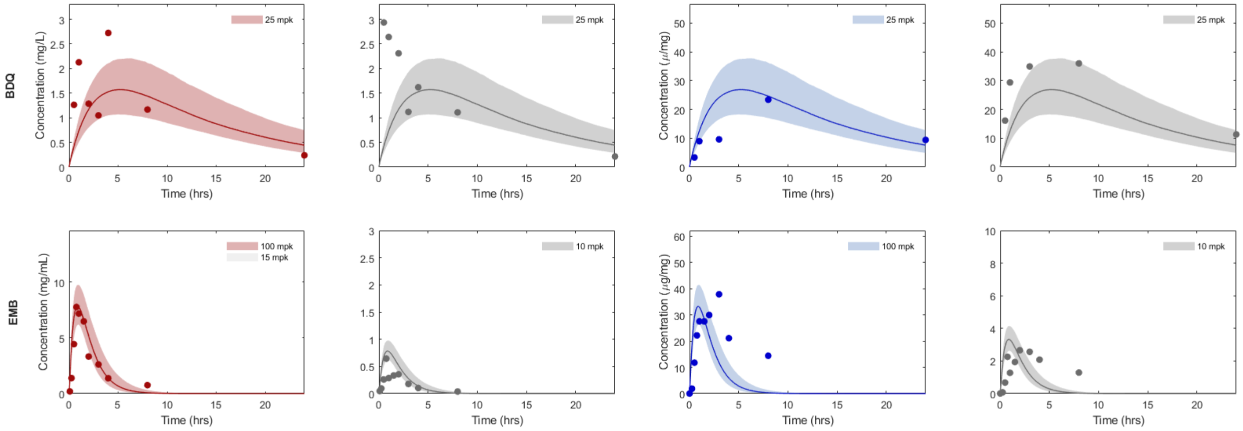

Supplementary Figure 4: Visual predictive check of the mPBPK model for bedaquiline (BDQ) and ethambutol (EMB) in plasma and lung. The figure shows the performance in training and validations for the mPBPK model in plasma and lung for all the doses. All figures show the median of the simulated VP (solid line) and the fifth and ninety-fifth quantiles (shaded area). Dots represent the experimental data. Red refers to the training sets in plasma, blue to the training sets in lungs, and grey are the validation sets.

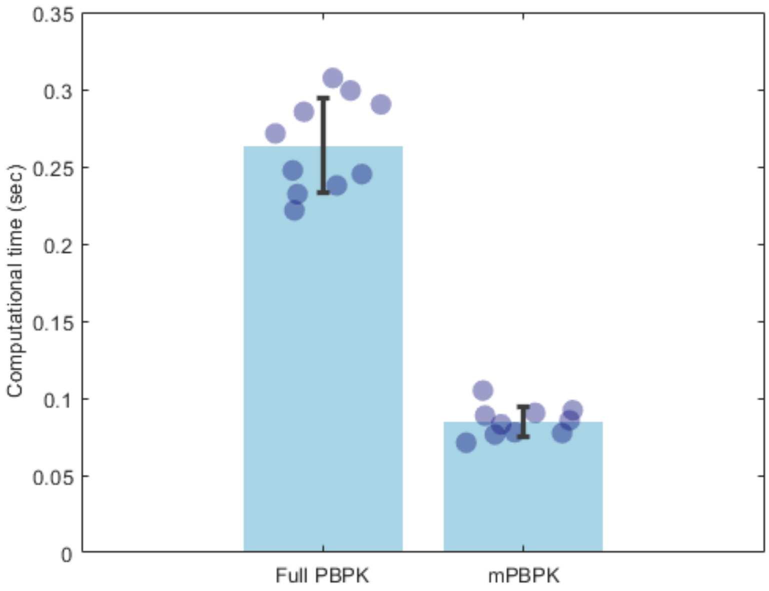

Supplementary Figure 5: Simulation time comparison between the mPBPK model and the reference full PBPK model. Points show the single runtime, bars the mean and standard deviation.

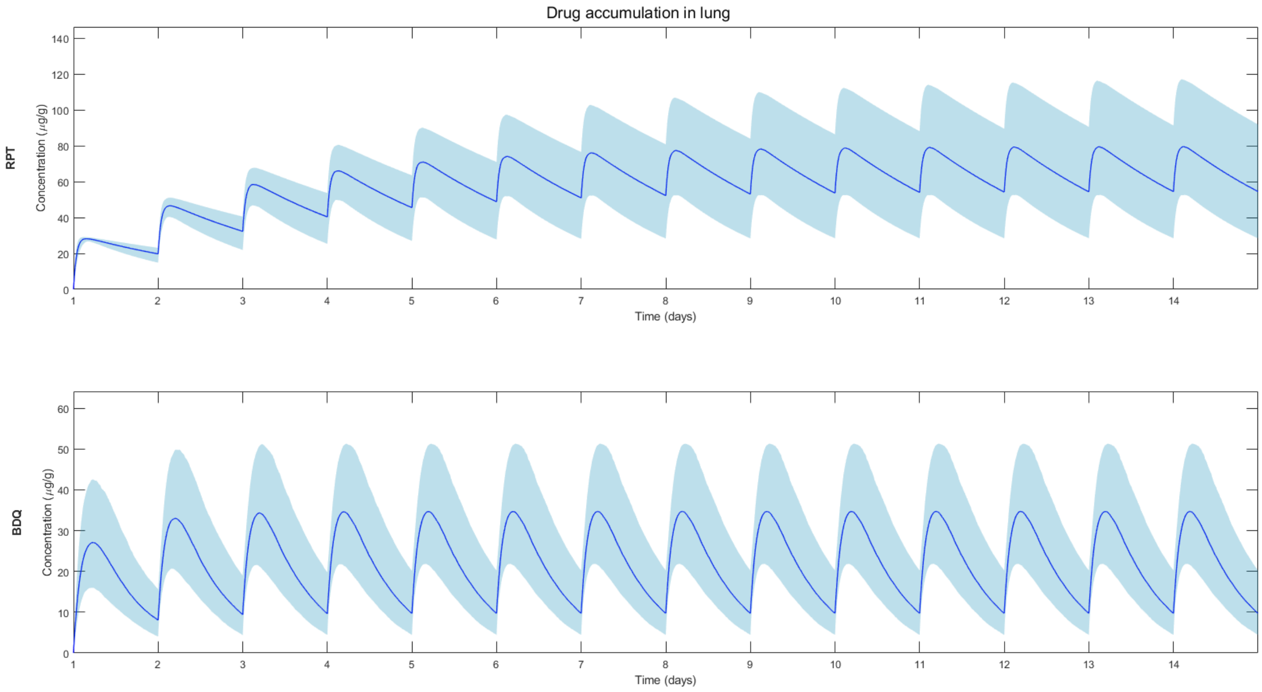

Supplementary Figure 6: PK dynamics in lungs for rifapentine (RPT, 20 mg/kg) and bedaquiline (BDQ, 25 mg/kg) at human equivalent doses. The model-based simulation is run for 14 days until the metabolic steady-state is reached.

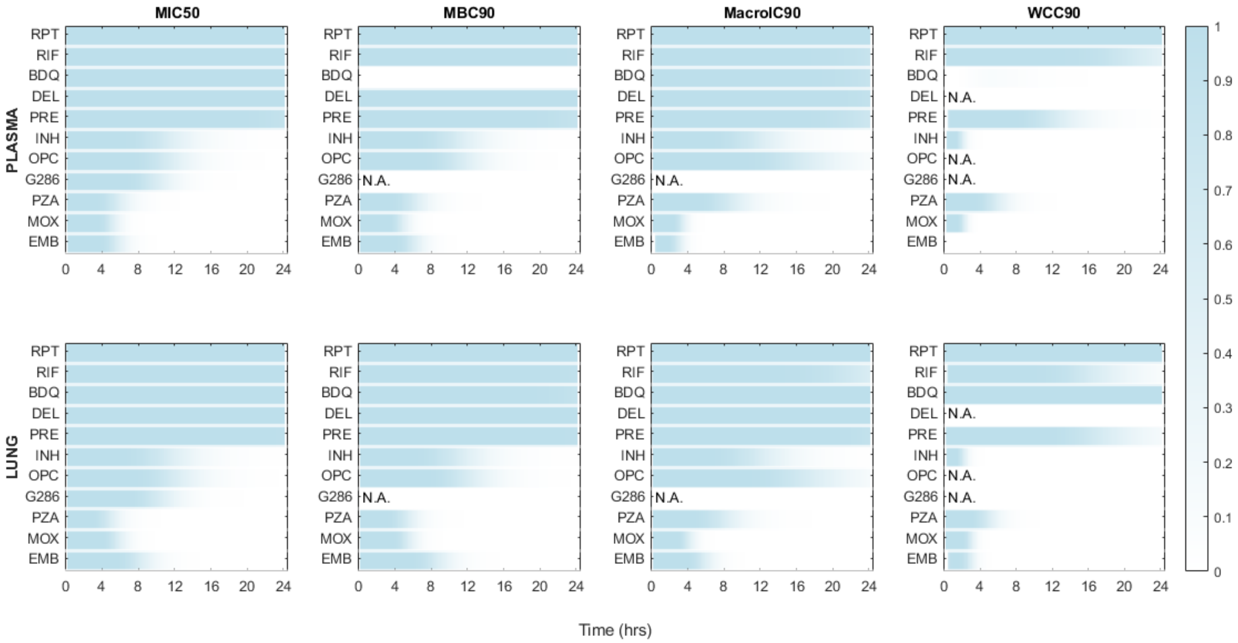

Supplementary Figure 7: Relative target attainment in plasma and lungs of all the compounds with reference human-equivalent doses (20 mg/kg RPT, 10 mg/kg RIF, 25 mg/kg BDQ, 3 mg/kg DEL, 100 mg/kg PRE, 25 mg/kg INH, 2.5 mg/kg OPC, 35 mg/kg G286, 150 mg/kg PZA, 100 mg/kg MOX, 100 mg/kg EMB). The color shades indicate the posterior probability of the VP of being above the threshold in the tile at the time indicated in the x-axis. NA: the metric value is not available. For GSK-2556286 only MIC90 was available and has been used in place of MIC50 for this analysis.

References

Ahmad, Z. *et al.* (2011) 'PA-824 exhibits time-dependent activity in a murine model of tuberculosis', *Antimicrobial Agents and Chemotherapy*, 55(1), pp. 239–245. Available at: https://doi.org/10.1128/AAC.00849-10.

Alfarisi, O. *et al.* (2017) 'Rifampin vs. rifapentine: what is the preferred rifamycin for tuberculosis?', *Expert Review of Clinical Pharmacology*, 10(10), pp. 1027–1036. Available at: https://doi.org/10.1080/17512433.2017.1366311.

Bigelow, K.M. *et al.* (2021) 'Pharmacodynamic Correlates of Linezolid Activity and Toxicity in Murine Models of Tuberculosis', *Journal of Infectious Diseases*, 223(11), pp. 1855–1864. Available at: https://doi.org/10.1093/infdis/jiaa016.

Bruzzese, T. *et al.* (2000) 'Pharmacokinetics and tissue distribution of rifametane, a new 3- azinomethyl-rifamycin derivative, in several animal species', *Arzneimittel-Forschung/Drug Research*, 50(1), pp. 60–71. Available at: https://doi.org/10.1055/s-0031-1300165.

Gaohua, L. *et al.* (2015) 'Development of a Multicompartment Permeability-Limited Lung PBPK Model and Its Application in Predicting Pulmonary Pharmacokinetics of Antituberculosis Drugs', *CPT: Pharmacometrics and Systems Pharmacology*, 4(10), pp. 605–613. Available at: https://doi.org/10.1002/psp4.12034.

Hariguchi, N. *et al.* (2020) 'OPC-167832, a novel carbostyril derivative with potent antituberculosis activity as a DPRE1 inhibitor', *Antimicrobial Agents and Chemotherapy*, 64(6). Available at: https://doi.org/10.1128/AAC.02020-19.

Hirao, Y. *et al.* (2015) 'Liquid Chromatography-Tandem Mass Spectrometry Methods for Determination of Delamanid in Mouse Plasma and Lung', *American Journal of Analytical Chemistry*, 6(2), pp. 98–105. Available at: https://doi.org/10.4236/AJAC.2015.62009.

Humphries, H. *et al.* (2021) 'Development of physiologically-based pharmacokinetic models for standard of care and newer tuberculosis drugs', *CPT: Pharmacometrics & Systems Pharmacology*, 10(11), p. 1382. Available at: https://doi.org/10.1002/PSP4.12707.

Irwin, S.M. *et al.* (2016) 'Bedaquiline and Pyrazinamide Treatment Responses Are Affected by Pulmonary Lesion Heterogeneity in Mycobacterium tuberculosis Infected C3HeB/FeJ Mice', *ACS Infectious Diseases*, 2(4), pp. 251–267. Available at: https://doi.org/10.1021/acsinfecdis.5b00127.

Jayaram, R. *et al.* (2004) 'Isoniazid pharmacokinetics-pharmacodynamics in an aerosol infection model of tuberculosis', *Antimicrobial Agents and Chemotherapy*, 48(8), pp. 2951–2957. Available at: https://doi.org/10.1128/AAC.48.8.2951-2957.2004.

Kaniga, K. *et al.* (2016) 'A Multilaboratory, Multicountry Study To Determine Bedaquiline MIC Quality Control Ranges for Phenotypic Drug Susceptibility Testing', *Journal of Clinical Microbiology*, 54(12), pp. 2956–2962. Available at: https://doi.org/10.1128/JCM.01123-16.

Lakshminarayana, S.B. *et al.* (2014) ‘Pharmacokinetics-pharmacodynamics analysis of bicyclic 4-nitroimidazole analogs in a murine model of tuberculosis’, *PLoS ONE*, 9(8). Available at: https://doi.org/10.1371/journal.pone.0105222.

Lakshminarayana, S.B. *et al.* (2015) ‘Comprehensive physicochemical, pharmacokinetic and activity profiling of anti-TB agents’, *Journal of Antimicrobial Chemotherapy*, 70(3), pp. 857–867. Available at: https://doi.org/10.1093/jac/dku457.

Litjens, C.H.C. *et al.* (2022) ‘Prediction of Moxifloxacin Concentrations in Tuberculosis Patient Populations by Physiologically Based Pharmacokinetic Modeling’, *The Journal of Clinical Pharmacology*, 62(3), pp. 385–396. Available at: https://doi.org/10.1002/jcph.1972.

Lyons, M.A. (2018) ‘Modeling and simulation of pretomanid pharmacokinetics in pulmonary tuberculosis patients’, *Antimicrobial Agents and Chemotherapy*, 62(7). Available at: https://doi.org/10.1128/AAC.02359-17.

Mudde, S.E. *et al.* (2022) ‘Delamanid or pretomanid? A Solomonic judgement!’, *Journal of Antimicrobial Chemotherapy*, 77(4), pp. 880–902. Available at: https://doi.org/10.1093/jac/dkab505.

Muliaditan, M. *et al.* (2022) ‘Prediction of lung exposure to anti-tubercular drugs using plasma pharmacokinetic data: Implications for dose selection’, *European Journal of Pharmaceutical Sciences*, 173(December 2021), p. 106163. Available at: https://doi.org/10.1016/j.ejps.2022.106163.

Muliaditan, M. and Della Pasqua, O. (2022) ‘Bacterial growth dynamics and pharmacokinetic–pharmacodynamic relationships of rifampicin and bedaquiline in BALB/c mice’, *British Journal of Pharmacology*, 179(6), pp. 1251–1263. Available at: https://doi.org/10.1111/BPH.15688.

Nuermberger, E. *et al.* (2006) ‘Combination chemotherapy with the nitroimidazopyran PA-824 and first-line drugs in a murine model of tuberculosis’, *Antimicrobial Agents and Chemotherapy*, 50(8), pp. 2621–2625. Available at: https://doi.org/10.1128/AAC.00451-06.

Nuermberger, E.L. *et al.* (2022) ‘GSK2556286 Is a Novel Antitubercular Drug Candidate Effective In Vivo with the Potential To Shorten Tuberculosis Treatment’, *Antimicrobial Agents and Chemotherapy*, 66(6). Available at: https://doi.org/10.1128/AAC.00132-22.

Robertson, G.T. *et al.* (2021) ‘Comparative analysis of pharmacodynamics in the c3heb/fej mouse tuberculosis model for DprE1 inhibitors TBA-7371, PBTZ169, and OPC-167832’, *Antimicrobial Agents and Chemotherapy*, 65(11). Available at: https://doi.org/10.1128/AAC.00583-21.

Rosenthal, I.M. *et al.* (2005) ‘Weekly moxifloxacin and rifapentine is more active than the Denver regimen in murine tuberculosis’, *American Journal of Respiratory and Critical Care Medicine*, 172(11), pp. 1457–1462. Available at: https://doi.org/10.1164/rccm.200507-1072OC.

Rosenthal, I.M. *et al.* (2007) ‘Daily dosing of rifapentine cures tuberculosis in three months or less in the murine model’, *PLoS Medicine*, 4(12), pp. 1931–1939. Available at: https://doi.org/10.1371/journal.pmed.0040344.

Sasahara, K. *et al.* (2015) ‘Pharmacokinetics and metabolism of delamanid, a novel anti-tuberculosis drug, in animals and humans: Importance of albumin metabolism in vivo’, *Drug Metabolism and Disposition*, 43(8), pp. 1267–1276. Available at: https://doi.org/10.1124/DMD.115.064527.

Shibata, M. *et al.* (2021) ‘Prediction of Human Pharmacokinetic Profiles of the Antituberculosis Drug Delamanid from Nonclinical Data: Potential Therapeutic Value against Extrapulmonary Tuberculosis.’, *Antimicrobial agents and chemotherapy*, 65(8), p. e0257120. Available at: https://doi.org/10.1128/AAC.02571-20.

Shimokawa, Y. *et al.* (2015) ‘Metabolic mechanism of delamanid, a new anti-tuberculosis drug, in human plasma’, *Drug Metabolism and Disposition*, 43(8), pp. 1277–1283. Available at: https://doi.org/10.1124/dmd.115.064550.

Strydom, N. *et al.* (2019) ‘Tuberculosis drugs’ distribution and emergence of resistance in patient’s lung lesions: A mechanistic model and tool for regimen and dose optimization’, *PLOS Medicine*. Edited by M. Murray, 16(4), p. e1002773. Available at: https://doi.org/10.1371/journal.pmed.1002773.

Wallace, R.J., Philley, J. V. and Griffith, D.E. (2015) ‘Antimycobacterial Agents’, in *Mandell, Douglas, and Bennett’s Principles and Practice of Infectious Diseases*. Elsevier, pp. 463-478.e3. Available at: https://doi.org/10.1016/B978-1-4557-4801-3.00038-2.

Yu, X. *et al.* (2016) ‘Wild-Type and Non-Wild-Type Mycobacterium tuberculosis MIC Distributions for the Novel Fluoroquinolone Antofloxacin Compared with Those for Ofloxacin, Levofloxacin, and Moxifloxacin’, *Antimicrobial Agents and Chemotherapy*, 60(9), pp. 5232–5237. Available at: https://doi.org/10.1128/AAC.00393-16.
